# Supplementary material for: FABP5 regulates ether lipid metabolism to ameliorate atopic dermatitis
Source: bioRxiv. 2025 Oct 25:2025.10.24.684205. Preprint. [Version 1] doi: 10.1101/2025.10.24.684205 (PMC12633262; doi:10.1101/2025.10.24.684205)
Supplement: 1 [file NIHPP2025.10.24.684205V1-supplement-1.pdf]

bioRxiv preprint doi: https://doi.org/10.1101/2025.10.24.684205; this version posted October 25, 2025. The copyright holder for this preprint (which was not certified by peer review) is the author/funder, who has granted bioRxiv a license to display the preprint in perpetuity. It is made available under aCC-BY 4.0 International license.

Supplementary Table 1

List of antibodies and their working dilutions for use in flow cytometry analysis

Antibody	Clone	Vendor Cat No.	Dilution
Anti-mouse CD45 AF700	30-F11	BioLegend 103128	1:400
Anti-mouse CD3e APC	145-2C11	BioLegend 100312	1:400
Anti-mouse CD19 APC	6D5	BioLegend 115512	1:400
Anti-mouse/human CD11b PB	M1/70	BioLegend 101224	1:400
Anti-mouse Ly6C BV605	HK1.4	BioLegend 128036	1:400
Anti-mouse Ly6G AF488	1A8	BioLegend 127626	1:400
Anti-mouse CD64 PE	X54-5/7.1	BioLegend 139304	1:400
Anti-mouse Epcam PerCPcy5.5	68.8	BioLegend 118220	1:400
Anti-mouse CD11c PEcy7	N418	BioLegend 117318	1:400
Anti-mouse Ly6G APCcy7	1A8	BioLegend 127624	1:400
Anti-mouse/human CD11b APCcy7	M1/70	BioLegend 101222	1:400
Anti-mouse CD3e AF700	500A2	BioLegend 152316	1:400
Anti-mouse TCRγδ FITC	GL3	BioLegend 118106	1:400
Anti-mouse TCRβ BV711	H57-597	BioLegend 109243	1:400
Anti-mouse CD8α PEcy7	53-6.7	BioLegend 100722	1:400
Anti-mouse CD4 BV605	RM4-5	BioLegend 100548	1:400
Anti-mouse CD45 BV711	30-F11	BioLegend 103147	1:400
Anti-mouse CD200R3 PE	Ba13	BioLegend 142205	1:400
Anti-mouse IgE BV421	R35-72	BD 564207	1:400
Anti-mouse cKit PEcy7	2B8	BioLegend 105814	1:400
Anti-mouse I-A/I-E APCcy7	M5/114.15.2	BioLegend 107628	1:800
Anti-mouse/human CD11b FITC	M1/70	BioLegend 101206	1:400
Anti-mouse IL-4 PE	11B11	BD 554435	1:400
Anti-mouse IFNγ BV421	XMG1.2	BioLegend 505830	1:400
Anti-mouse IL-17A AF647	TC11-18H10	BD 560184	1:400
Anti-HA.11 Epitope Tag AF647	16B12	BioLegend 682404	1:500
eBioscience™ Fixable Viability Dye eF506		Invitrogen 65-0866-14	1:1000

bioRxiv preprint doi: https://doi.org/10.1101/2025.10.24.684205; this version posted October 25, 2025. The copyright holder for this preprint (which was not certified by peer review) is the author/funder, who has granted bioRxiv a license to display the preprint in perpetuity. It is made available under aCC-BY 4.0 International license.

Figure S1:

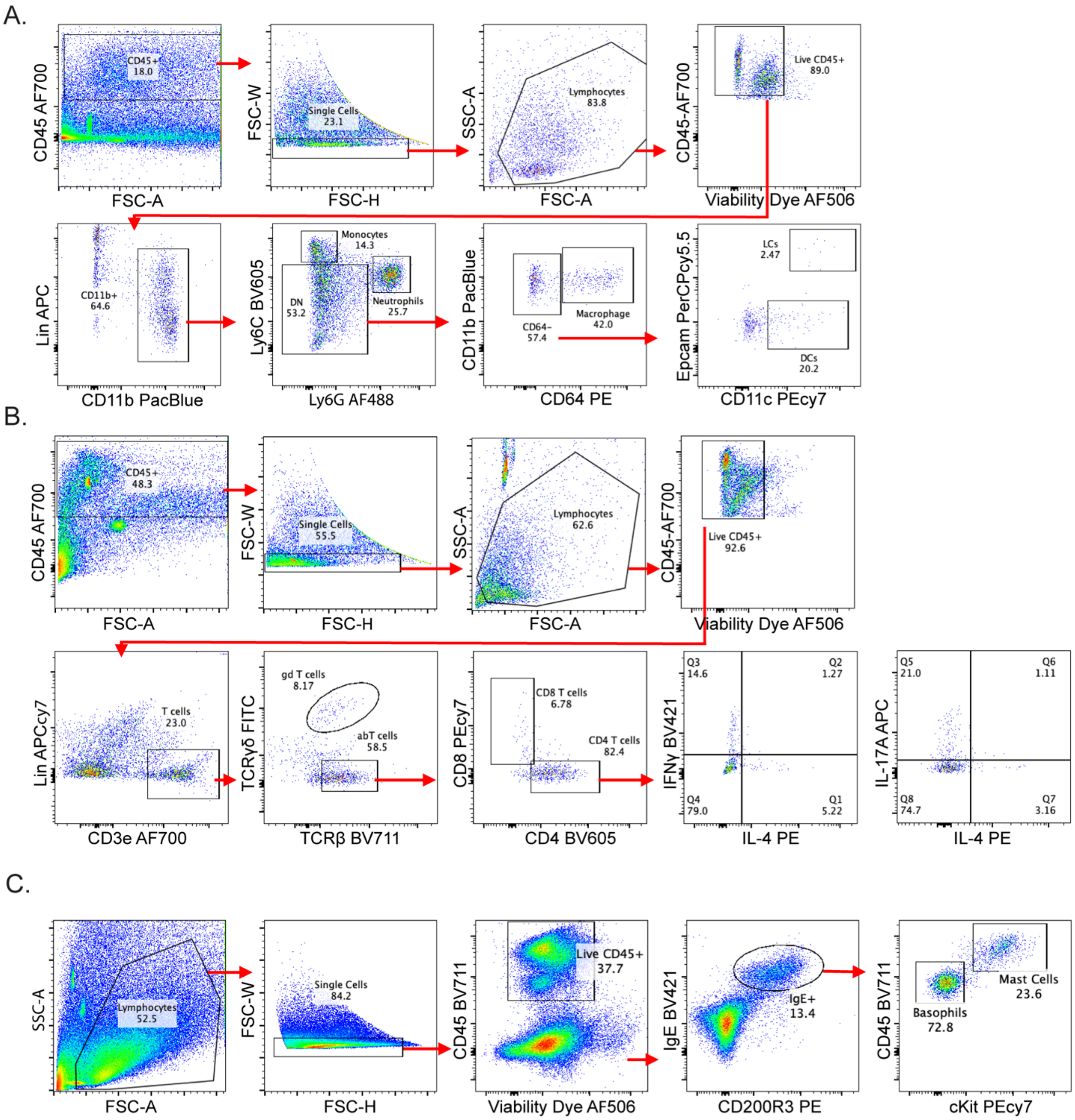

Supplemental Figure 1

A) Flow cytometry gating strategy for quantification of total CD45+ immune cells, monocytes, neutrophils, macrophages, dendritic cells (DCs) and Langerhans cells (LCs) isolated from the skin of MC903-treated mice.
B) Flow cytometry gating strategy for quantification of $CD4^{+}$ and $CD8^{+}$ T cells as well as the different cytokine secreting CD4+ T cell populations from the skin of MC903-treated mice.

bioRxiv preprint doi: https://doi.org/10.1101/2025.10.24.684205; this version posted October 25, 2025. The copyright holder for this preprint (which was not certified by peer review) is the author/funder, who has granted bioRxiv a license to display the preprint in perpetuity. It is made available under aCC-BY 4.0 International license.

C) Flow cytometry gating strategy for quantification of basophils and mast cells from the skin of MC903-treated mice.

bioRxiv preprint doi: https://doi.org/10.1101/2025.10.24.684205; this version posted October 25, 2025. The copyright holder for this preprint (which was not certified by peer review) is the author/funder, who has granted bioRxiv a license to display the preprint in perpetuity. It is made available under aCC-BY 4.0 International license.

Figure S2:

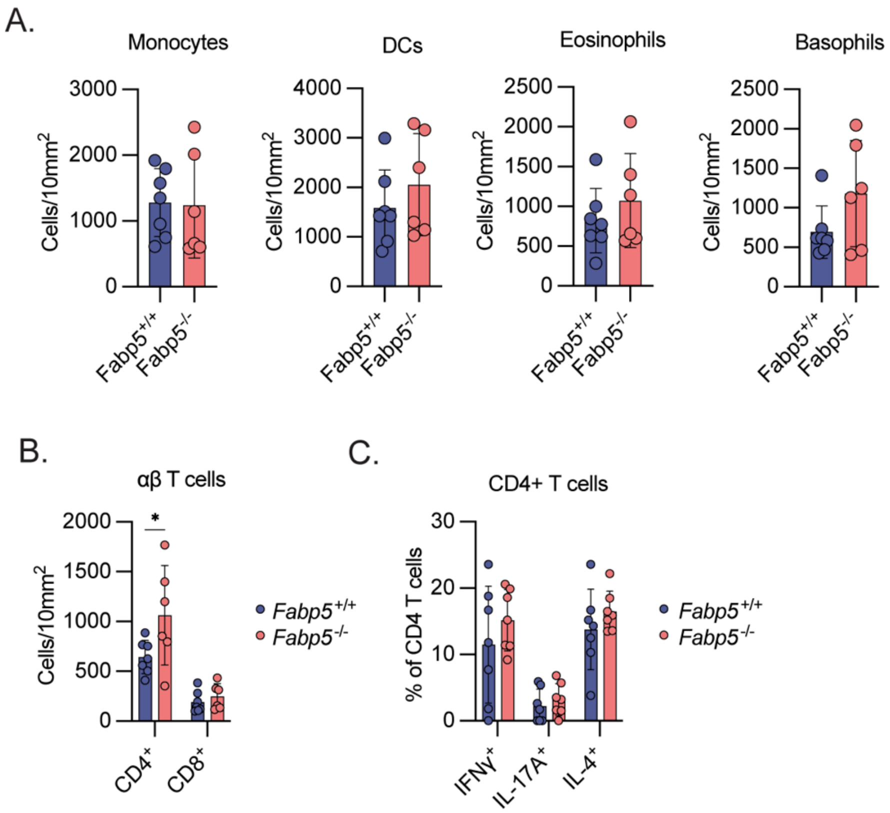

Supplemental Figure 2

A) Flow cytometry-based quantification of total monocytes, dendritic cells (DCs), eosinophils, and basophils in skin of *Fabp5*$^{+/+}$ and *Fabp5*$^{-/-}$ mice on day 10 of treatment with MC903. Cell count is normalized per unit area. (n = 13)

B) Flow cytometry-based quantification of CD4+ and CD8+ αβ T cells in skin of *Fabp5*$^{+/+}$ and *Fabp5*$^{-/-}$ mice on day 10 of treatment with MC903. Cell count is normalized per unit area. (n = 13)

C) Flow cytometry-based quantification of intracellular cytokine staining of CD4^{+} T cells in skin of *Fabp5*$^{+/+}$ and *Fabp5*$^{-/-}$ mice on day 10 of treatment with MC903. Cell count is normalized as percent of total CD4^{+} T cells (n = 14)

Unless otherwise noted, all data are reported as means ± SD. * p < 0.05; ** p < 0.01, *** p < 0.005. Two-tailed unpaired Student's t-test. Datapoints are discrete biological replicates. (C) and (D) were analyzed by one-way ANOVA with Fisher's least significant difference test.

bioRxiv preprint doi: https://doi.org/10.1101/2025.10.24.684205; this version posted October 25, 2025. The copyright holder for this preprint (which was not certified by peer review) is the author/funder, who has granted bioRxiv a license to display the preprint in perpetuity. It is made available under aCC-BY 4.0 International license.

Figure S3:

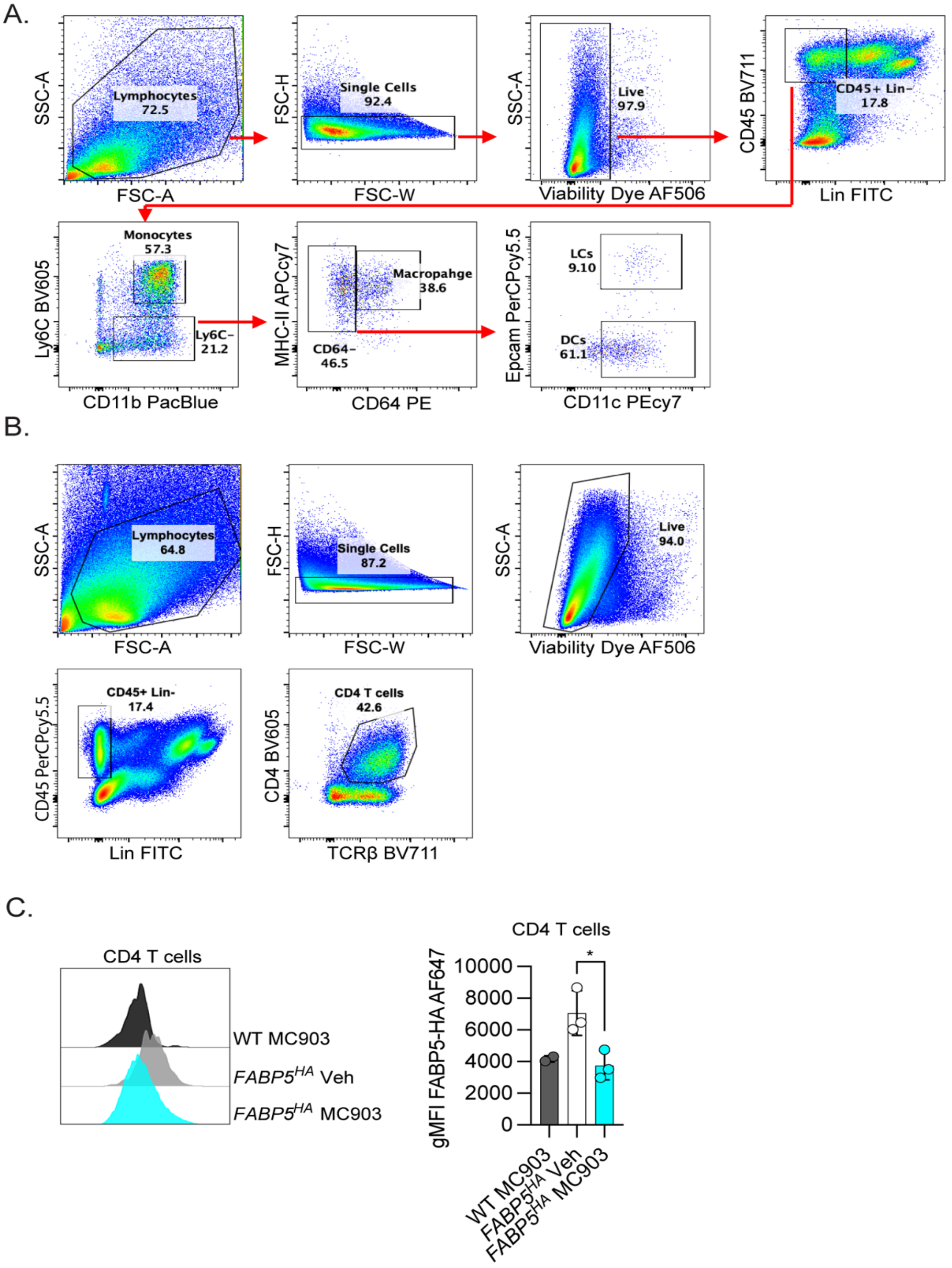

Supplemental Figure 3:

A) Flow cytometry gating strategy for identification of monocytes, macrophages, dendritic cells (DCs) and Langerhans cells (LCs) for quantification of intracellular FABP5-HA AF647 fluorescent intensity related to figure 2F.

bioRxiv preprint doi: https://doi.org/10.1101/2025.10.24.684205; this version posted October 25, 2025. The copyright holder for this preprint (which was not certified by peer review) is the author/funder, who has granted bioRxiv a license to display the preprint in perpetuity. It is made available under aCC-BY 4.0 International license.

B) Flow cytometry gating strategy for identification of $CD4^+$ T cells for quantification of intracellular FABP5-HA AF647 fluorescent intensity related to S3C.
C) Histogram (left) comparing the fluorescent intensity of intracellular α-HA AF647 staining in different $CD4^+$ T cells isolated from skin of WT and $FABP5^{HA}$ mice treated with vehicle or MC903. Quantification of geometric mean fluorescent intensity (gMFI) (right) from these same populations (n = 12).

Unless otherwise noted, all data are reported as means ± SD. * $p < 0.05$; ** $p < 0.01$, *** $p < 0.005$. Two-tailed unpaired Student's *t*-test. Datapoints are discrete biological replicates. (C) and (D) were analyzed by one-way ANOVA with Fisher's least significant difference test.

bioRxiv preprint doi: https://doi.org/10.1101/2025.10.24.684205; this version posted October 25, 2025. The copyright holder for this preprint (which was not certified by peer review) is the author/funder, who has granted bioRxiv a license to display the preprint in perpetuity. It is made available under aCC-BY 4.0 International license.

Figure S4:

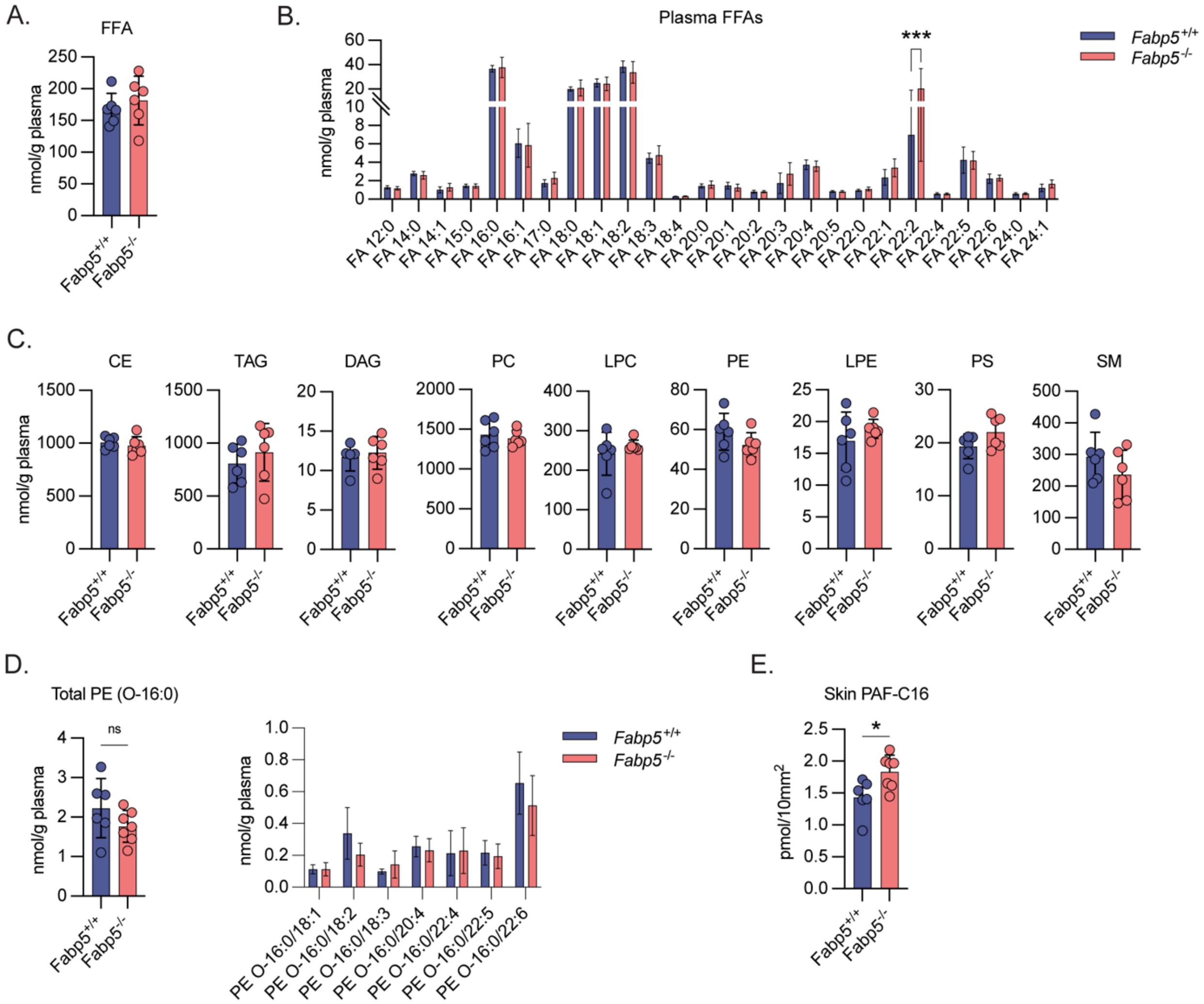

Supplemental Figure 4:

A) HPLC-MS/MS based quantification of total free fatty acids (FFA) in plasma of *Fabp5*$^{+/+}$ and *Fabp5*$^{-/-}$ mice on day 10 of treatment with MC903.
B) HPLC-MS/MS based quantification of individual FFA species in plasma of *Fabp5*$^{+/+}$ and *Fabp5*$^{-/-}$ mice on day 10 of treatment with MC903.
C) HPLC-MS/MS based quantification of total cholesterol ester (CE), triacylglycerol (TAG), diacylglycerol (DAG), phosphatidylcholine (PC), lysophosphatidylcholine (LPC), phosphatidylethanolamine (PE) lysophosphatidylethanolamine (LPE), phosphatidylserine (PS), and sphingomyelin (SM) species in plasma of *Fabp5*$^{+/+}$ and *Fabp5*$^{-/-}$ mice on day 10 of treatment with MC903.
D) HPLC-MS/MS based quantification of total PE species with 16:0 ether-linked tails (left) and quantification of individual PE species with 16:0 ether-linked tails (right) in plasma of *Fabp5*$^{+/+}$ and *Fabp5*$^{-/-}$ mice on day 10 of treatment with MC903.
E) Targeted LC-MS/MS based quantification of PAF C-16 from skin of *Fabp5*$^{+/+}$ and *Fabp5*$^{-/-}$ mice on day 10 of treatment with MC903.

Unless otherwise noted, all data are reported as means ± SD. * $p < 0.05$; ** $p < 0.01$, *** $p < 0.005$. Two-tailed unpaired Student's *t*-test. Datapoints are discrete biological replicates. (B) and (D) were analyzed by two-way ANOVA with Šidák correction for multiple comparisons.

bioRxiv preprint doi: https://doi.org/10.1101/2025.10.24.684205; this version posted October 25, 2025. The copyright holder for this preprint (which was not certified by peer review) is the author/funder, who has granted bioRxiv a license to display the preprint in perpetuity. It is made available under aCC-BY 4.0 International license.

Figure S5:

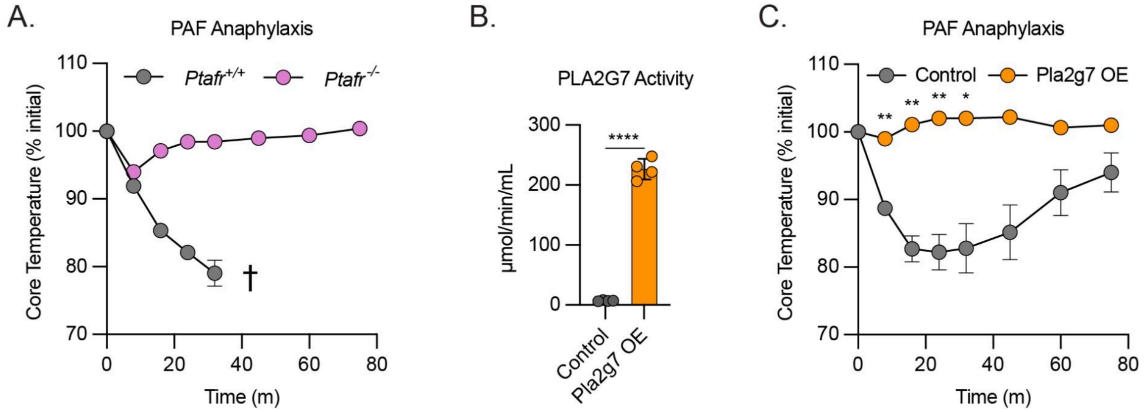

Supplemental Figure 5:

A) Core body temperatures (% of initial) following PAF induced anaphylaxis in naïve *Ptafr*$^{+/+}$ and *Ptafr*$^{-/-}$ mice. (n = 6). Dots show mean ± SEM. Stars represent Šidák corrected *p* values for 2-way ANOVA with multiple comparisons.

B) PLA2G7 enzymatic activity on 2-thio PAF from serum of mice one day following hydrodynamic injection with control plasmid or plasmid encoding Pla2g7 (PLA2G7 OE). (n = 8)

C) Core body temperatures (% of initial) following PAF induced anaphylaxis in mice one day following hydrodynamic injection with plasmid encoding Pla2g7 (PLA2G7 OE) or equal volume saline control. (n = 8). Dots show mean ± SEM. Stars represent Šidák corrected *p* values for 2-way ANOVA with multiple comparisons.

Unless otherwise noted, all data are reported as means ± SD. * $p < 0.05$; ** $p < 0.01$, *** $p < 0.005$. Two-tailed unpaired Student's *t*-test. Datapoints are discrete biological replicates.
